# Good Laboratory Practices: Becker et al. Respond

**DOI:** 10.1289/ehp.0901755R

**Published:** 2010-05

**Authors:** Richard A. Becker, Erik R. Janus, Russell D. White, Francis H. Kruszewski, Robert E. Brackett

**Affiliations:** American Chemistry Council, Arlington, Virginia, E-mail: rick_becker@americanchemistry.com; Crop Life America, Washington, DC; American Petroleum Institute, Washington, DC; Soap and Detergent Association, Washington, DC; Grocery Manufacturers Association, Washington, DC

We appreciate the dialogue stimulated by our letter to the editor (Becker et al. 2009). Our intent was to respond only to Myers et al. (2009) regarding the purpose and function of Good Laboratory Practices (GLP) for weighting reliability of studies. Tyl (2009), in response to Myers et al. (2009), provided extensive point-by-point discussion of the specific studies.

In his letter, Tweedale implies that we argued to *a priori* exclude academic, non-GLP studies from risk assessments. To the contrary, we clearly stated that “[e]ach study, GLP and non-GLP, should be evaluated and weighed in accordance with fundamental scientific principles” (Becker et al. 2009). We fully agree with Tweedale that sources of funding should be disclosed, that researchers should “make their methods and data more freely available,” and more industry-supported studies should be published in scientific journals. With respect to bias, Maurissen et al. (2005) and Barrow and Conrad (2006) discussed the spectrum of mechanisms in place to ensure the integrity of industry-sponsored research. Ultimately, all scientific research must stand on its merits. However, it is unscientific to eliminate or devalue any study based solely on the organization that conducted the study, the affiliation of an investigator, or the source of funding. The Society of Toxicology (2008) has stated this principle quite clearly: “[r]esearch should be judged on the basis of scientific merit, without regard for the funding source or where the studies are conducted (e.g., academia, government, or industry).”

Moreover, we did not seek to call into question scientific journal peer review per se, but instead to point out that whereas all study records and data from GLP investigations are available to regulatory agencies, rarely are such details made available as part of a peer-reviewed article published in a scientific journal. The point we wish to emphasize is that typical regulatory safety assessment studies conducted in accordance with GLP *a*) must follow agency test guidelines to assure use of relevant test systems, sufficient and applicable dosing protocols, and adequate dose groups and sizes, and *b*) must evaluate specific end points that regulatory organizations consider validated. Further, such GLP studies submitted to regulatory agencies generally include both a full study report and all raw data. This level of scientific rigor and the extensive data of a GLP study allow a regulatory agency to conduct a comprehensive review and to reach a fully independent conclusion. For these reasons, greater weight and confidence are generally afforded to GLP studies. Now, with the increasingly common practice of journals providing access to supplemental data, there are expanded opportunities for researchers to disseminate actual study data; this should facilitate independent evaluation by regulatory agencies.

As scientists specializing in regulatory safety evaluations, we have extensive experience in interpreting chemical toxicity studies from government, academia, and private- sector laboratories. In conducting chemical risk assessments, we believe that scientists from all sectors should support the use of objective criteria for determining data quality and study reliability (Schneider et al. 2009) coupled with a structured evaluative framework, such as that of the World Health Organization International Programme on Chemical Safety (Boobis et al. 2006, 2008), to provide a systematic approach for assessing the overall weight of the evidence for observed effects and the postulated mode of action. In this manner, data from laboratory experiments, epidemiological investigations, and cutting-edge mechanistic research from all relevant studies—GLP and non-GLP—and from all investigators, regardless of affiliation or funding source, can be comprehensively reviewed, given appropriate weight, and integrated in a manner that provides a robust, biologically plausible understanding of the potential hazards and risks that exposures to a substance could pose.
